# Using implementation science for WHO noncommunicable disease technical packages in low-and middle-income countries: a scoping review

**DOI:** 10.3389/frhs.2026.1816601

**Published:** 2026-07-30

**Authors:** Ishu Kataria, Lillian Morrell, Gavin Allman, Giulia Loffreda, Tiina Laatikainen, J. Jaime Miranda, Yodi Mahendradhata, Rachel Nugent

**Affiliations:** 1 RTI International, New Delhi, India; 2 Institute of Cancer Policy, King’s College London, London, United Kingdom; 3 RTI International, Durham, NC, United States; 4 Alliance for Health Policy and Systems Research, WHO, Geneva, Switzerland; 5 School of Medicine, University of Eastern Finland, Kuopio, Finland; 6 CRONICAS Centre of Excellence in Chronic Diseases, Universidad Peruana Cayetano Heredia, Lima, Peru; 7 Sydney School of Public Health, Faculty of Medicine and Health, The University of Sydney, Sydney, NSW, Australia; 8 Faculty of Medicine, Public Health and Nursing, Universitas Gadjah Mada, Yogyakarta, Indonesia; 9 University of Washington, Seattle, WA, United States

**Keywords:** ERIC, implementation science, LMICs, noncommunicable diseases, WHO technical package

## Abstract

**Introduction:**

The World Health Organization (WHO) created technical packages to support countries in preventing and treating noncommunicable diseases (NCDs). While these packages provide evidence-informed guidance for prevention, detection, and management of NCDs, little is known about how implementation science concepts and methods have been used in their adoption and scale-up in low- and middle-income countries (LMICs). This scoping review examines how implementation and dissemination science have been used across WHO NCD technical packages and the extent to which these efforts align with national NCD strategies.

**Methods:**

We conducted a scoping review of peer-reviewed literature (2009–2023) on the implementation or adaptation of WHO technical packages for the prevention and control of NCDs, in LMICs. Eight databases were searched. Data were extracted on use of implementation frameworks, barriers and facilitators, stakeholder engagement, capacity-building strategies, implementation outcomes, and sustainability. Findings were organized using domains aligned with the Expert Recommendations for Implementing Change (ERIC) framework.

**Results:**

and discussion: Fifty-seven studies from 27 LMICs met inclusion criteria. Most examined PEN or PEN-Plus, followed by HEARTS and MPOWER. Only one study explicitly applied a formal implementation science framework, and few explicitly reported using a conceptual mode or framework to guide implementation or evaluation. Stakeholder engagement was described inconsistently and mostly limited to providers and programme managers. Barriers reported commonly related to limited medicines, diagnostics, workforce capacity, and weak information systems, while facilitators included standardized protocols, team-based care, and use of decision-support tools. Capacity-building efforts centred on training, tools, and technical assistance, with minimal use of incentives. Implementation outcomes were primarily measured at the patient or facility level, whereas sustainability was rarely assessed. Few studies examined links between technical package implementation and national NCD action plans or monitoring frameworks. Despite growing uptake of WHO technical packages in LMICs, implementation science remains underutilized and applied inconsistently. Limited use of formal frameworks, weak integration with national policy processes, and scarce assessment of sustainability constrain the implementation and adaptation of these packages to deliver long-term impact. Strengthening structured implementation approaches, investing in research and system capacity, and embedding implementation science within policy cycles are critical to accelerating and sustaining progress on NCDs in LMICs.

## Introduction

Recognizing the growing burden of noncommunicable diseases (NCDs) in low- and middle-income countries (LMICs), the World Health Organization (WHO) has developed a suite of technical packages that provide evidence-informed guidance and tools to strengthen national responses. These packages support countries in implementing cost-effective interventions across prevention, early detection, treatment, and management. Among them, the WHO Package of Essential Noncommunicable Disease Interventions (WHO PEN; 2010) is the most widely adopted, defining core primary care interventions for cardiovascular diseases, diabetes, and chronic respiratory diseases, while incorporating healthy lifestyle promotion, self-care, palliative care, and training tools (1). Other technical packages, such as MPOWER, HEARTS, SHAKE, SAFER, and REPLACE, span both regulatory and clinical domains, addressing tobacco use, cardiovascular risk management, salt reduction, alcohol control, and trans-fat elimination (2–6). Collectively, these tools contribute to comprehensive national NCD action plans by guiding population-level policy reforms and strengthening frontline service delivery, while allowing countries the flexibility to adapt them to local needs, capacities, and health system contexts (3).

Despite global progress, many LMICs remain off-track to meet the NCD targets set in the Sustainable Development Goals (7). Multiple macro-, meso-, and micro-level health system challenges including shortages of essential medicines and diagnostics, insufficient workforce capacity, weak data systems, fragmented financing arrangements, and constrained fiscal space continue to hinder effective adoption and implementation of policies (8). Implementation science, encompassing both dissemination and implementation approaches, plays a critical role in navigating these challenges (9, 10). It helps countries adapt WHO technical packages by disseminating best practices, identifying barriers and facilitators, and evaluating implementation effectiveness across diverse contexts (11–13).

Because WHO technical packages include both clinical protocols and population-level policy measures, implementation challenges span facility workflows, quality of care, governance processes, and regulatory enforcement areas where implementation science offers significant value. With robust implementation science, national authorities can learn from global experience, tailor WHO guidance to local health system capabilities, and strengthen planning for sustainable policy and service delivery integration. Context-specific barriers and facilitators can be systematically addressed, supporting smooth implementation, while global evidence on effectiveness and cost-effectiveness can inform prioritization and resource allocation. This paper summarizes the current body of research on the use of implementation science in applying WHO technical packages, describing commonly used frameworks and processes, highlighting best practices, identifying persistent challenges, and outlining future research needs. Its objectives are to examine how implementation science supports adoption, implementation, and evaluation of WHO technical packages; identify frequently used implementation frameworks and strategies; explore barriers and facilitators; and assess how these efforts align with national NCD action plans and broader health system priorities.

## Methods

This scoping review examined peer-reviewed literature published between January 2009 and March 2023 that discussed the implementation or dissemination of at least one WHO technical package for NCDs in LMICs. In line with Peters' definition of implementation science (2013), we included studies that focused on the design, adaptation, or evaluation of the NCD policies within one or more of these packages (14). We included publications on the following WHO technical packages: PEN, PEN-Plus, HEARTS, MPOWER, SHAKE, SAFER, and REPLACE. The recommendations within these packages range from regulatory, population-level, and policy levers to interventions in service delivery and facility-based care, and studies of any package were eligible if they addressed factors that affect the adoption, fidelity, scale-up, or sustainability of WHO guidance implementation. We excluded systematic reviews, protocols, commentaries, non-English publications, studies for which full text was not available, and studies conducted exclusively in high-income countries. Global and multi-country studies were eligible if they included one or more low- or middle-income countries and reported findings relevant to LMIC implementation of WHO NCD technical packages.

### Search strategy

We conducted a literature search of the following databases for studies published on or after January 1, 2009 (date when the first WHO NCD package was introduced) and before March 2023: PubMed, PsycINFO, Cochrane Library, CINAHL, Web of Science, Embase, Global Health Database, and OpenDOAR. Search strings combined terms for LMICs, implementation or dissemination processes, and the names of WHO technical packages (e.g., “*PEN” OR “HEARTS” OR “MPOWER” OR “SHAKE” OR “SAFER” OR “REPLACE”* AND “*implementation science” OR “policy analysis” OR “monitoring” OR “evaluation”*). One reviewer conducted the electronic database searches using a predefined search strategy, and two reviewers (GA, LM) independently screened the resulting titles, abstracts, and full texts to determine whether they met the inclusion criteria, using a combination of the Rayyan web tool and Microsoft Excel. Two additional reviewers (IK, RN) resolved differences. The complete electronic search strategy for PubMed is provided in Supplement S1. The PubMed search strategy was adapted for use in the remaining databases by applying the equivalent controlled vocabulary and database-specific syntax.

### Data extraction

Two reviewers (GA and LM) independently extracted data using an Excel-based matrix. The matrix captured four levels of information: (1) bibliometric and descriptive details, including authorship, year of publication, country, relevant WHO technical package(s), and funding source; (2) implementation content, such as the frameworks applied, reported barriers and facilitators, stage of implementation (pre-, during, or post-implementation), and health-system capacity elements addressed; (3) strategies and structures, encompassing training and supervision mechanisms, technical assistance, tools, incentives, assessment or feedback processes, and modes of delivery; and (4) outcomes, covering patient-, provider-, system-, or policy-level effects, as well as sustainability or dissemination outcomes where these were reported.

### Data analysis and synthesis

We used the ERIC framework (15) as a guiding structure for organizing and interpreting implementation-related findings. The ERIC framework categorizes implementation strategies into conceptually related clusters, providing a standardized taxonomy for understanding how interventions are introduced, supported, and sustained within health systems. We selected the ERIC framework because the primary objective of this review was to identify and compare the implementation strategies used to support the adoption and delivery of WHO NCD technical packages across diverse LMIC settings. In contrast, CFIR primarily identifies determinants of implementation, EPIS conceptualises implementation across its exploration, preparation, implementation, and sustainment phases, RE-AIM provides a framework for evaluating implementation and maintenance, and Proctor's Implementation Outcomes Framework defines key implementation outcomes rather than implementation strategies. Because our objective was to synthesise the implementation strategies employed, rather than implementation determinants or outcomes, ERIC provided the most appropriate analytical framework.

To facilitate synthesis across diverse study designs and WHO technical packages, we developed five analytic domains based on established implementation science constructs. In line with established constructs in implementation science, five core domains were selected to capture the breadth of implementation processes: stakeholder engagement, assessment of barriers and facilitators, health system capacity, implementation outcomes, and sustainability. These analytic categories were developed to facilitate synthesis across heterogeneous studies rather than to replicate the ERIC taxonomy. They reflect key implementation science constructs and were subsequently mapped to the most relevant ERIC strategy clusters to enable consistent comparison across studies.

Extracted data from each study were reviewed and synthesized within these domains and subsequently mapped to the corresponding ERIC clusters to ensure conceptual consistency and facilitate cross-study comparison (Table 1). Implementation strategies described in each study were mapped to the ERIC taxonomy based on the reported intervention characteristics, while barriers, facilitators, implementation outcomes, and sustainability considerations were synthesized within the five analytic domains and interpreted alongside the corresponding ERIC strategy clusters. Following independent data extraction by GA and LM, one author (IK) reviewed the extracted implementation data and mapped the reported implementation strategies to the ERIC taxonomy using the published ERIC strategy definitions as a coding guide. Where studies described multiple implementation activities, more than one ERIC strategy or strategy cluster was assigned when appropriate. Any uncertainties in classification were resolved through discussion with the review team until consensus was reached.

**Table 1:** Mapping of key implementation components for wHO NCD technical packages to the ERIC framework.

Implementation Component	Relevant ERIC Strategy Cluster(s)	Representative ERIC Strategies
Stakeholder engagement	Develop stakeholder interrelationships; Utilize policy and organizational levers	Build a coalition; Identify and prepare champions; Use advisory boards and workgroups; Engage policymakers; Involve patients and communities
Barrier and facilitator assessment	Use evaluative and iterative strategies	Assess readiness and identify barriers and facilitators; Conduct local needs assessment; Obtain and use stakeholder feedback; Audit and provide feedback
Health system capacity assessment	Change infrastructure	Assess readiness and capacity; Conduct local needs assessment; Revise professional roles; Provide technical assistance; Facilitation
Implementation outcomes	Use evaluative and iterative strategies/Evaluate outcomes	Measuring effects of implementation to inform refinement and scale-up.
Sustainability	Plan for sustainability; Develop formal implementation blueprints; Change infrastructure	Plan for sustainability early; Institutionalize the intervention; Transition ownership to local champions; Maintain communication among implementers

Beyond documenting the interventions implemented, we extracted a distinct data element capturing the frameworks or conceptual models used to guide implementation, adaptation, or evaluation. These included frameworks such as CFIR, RE-AIM, EPIS, and other implementation science models where reported. Interventions were defined as specific actions, programmes, or policy measures, while frameworks referred to the models informing how these actions were delivered and assessed. Lastly, we analysed the geographic distribution of the included studies and the timing of the study relative to the stage of technical package implementation (i.e., research conducted before, during or after implementation of the guidelines in the study's geographic context).

Overall, we conducted a narrative synthesis of extracted data and used thematic comparative analysis to provide a descriptive summary for each paper. As WHO technical packages span both clinical and regulatory interventions, we applied the same implementation science domains across all packages, while interpreting the ERIC clusters in ways appropriate to each type of intervention (e.g., policy adoption and enforcement for regulatory packages vs. workflow change and service delivery for clinical packages). A formal critical appraisal of included studies was not conducted because the purpose of this scoping review was to map the breadth and characteristics of the available evidence rather than to evaluate methodological quality or determine the effectiveness of interventions. This approach is consistent with established guidance for scoping reviews.

## Results

**Figure 1:**
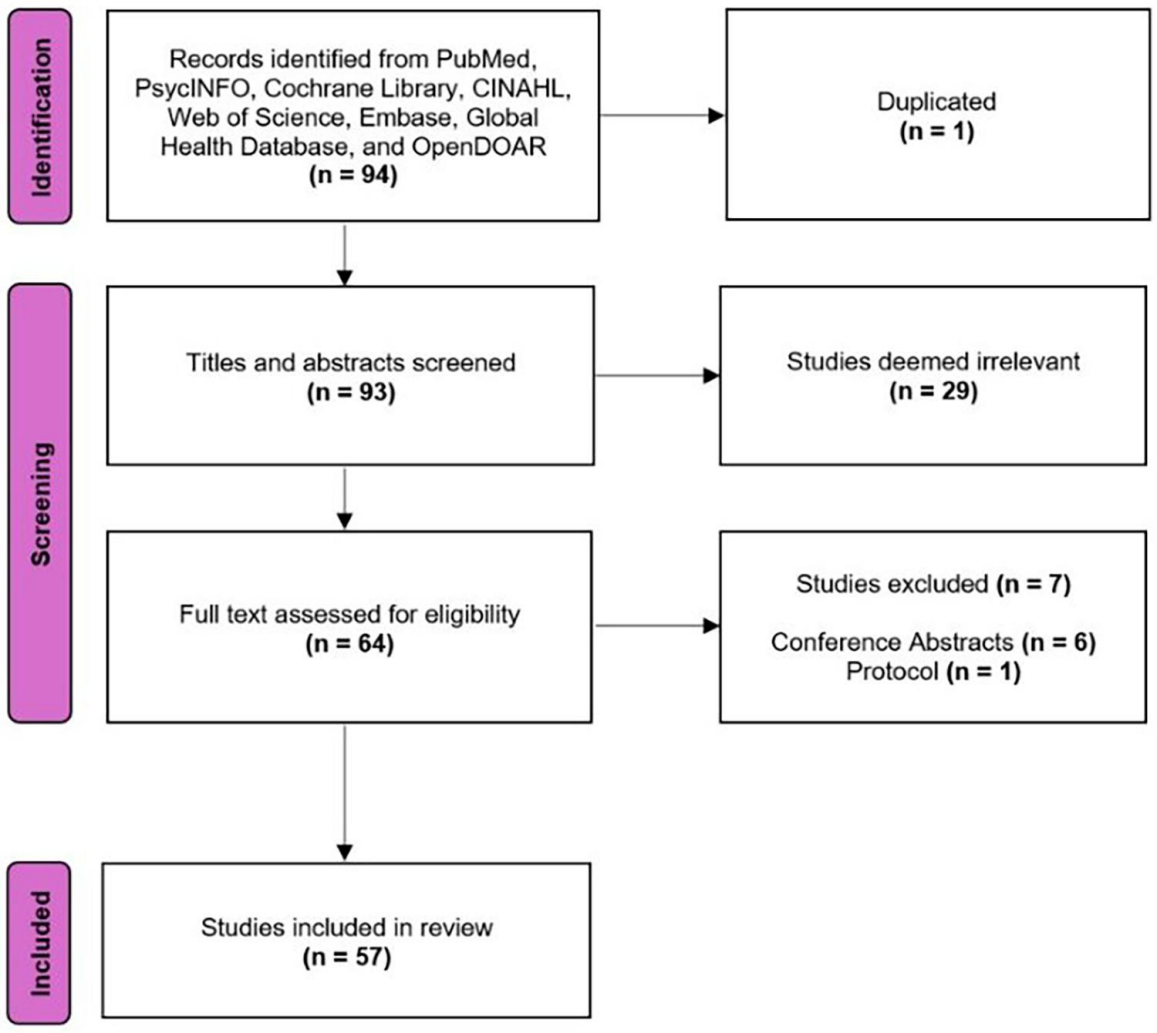
PRISMA-SCR flow diagram of study selection.

The search identified 93 studies, after excluding one duplicate search result. A total of 57 studies met the eligibility criteria and were included for data extraction and analysis (Figure 1).

This review included studies conducted in 27 countries representing all WHO regions. Studies from South East Asia were the most common, with the plurality in the region coming from India (*n* = 3) and Bhutan (*n* = 3). Two articles were from low-income countries, 21 from lower-middle income countries, and 14 from upper-middle-income countries as per the World Bank classification. We identified 20 multi-country studies from LMICs.

**Figure 2:**
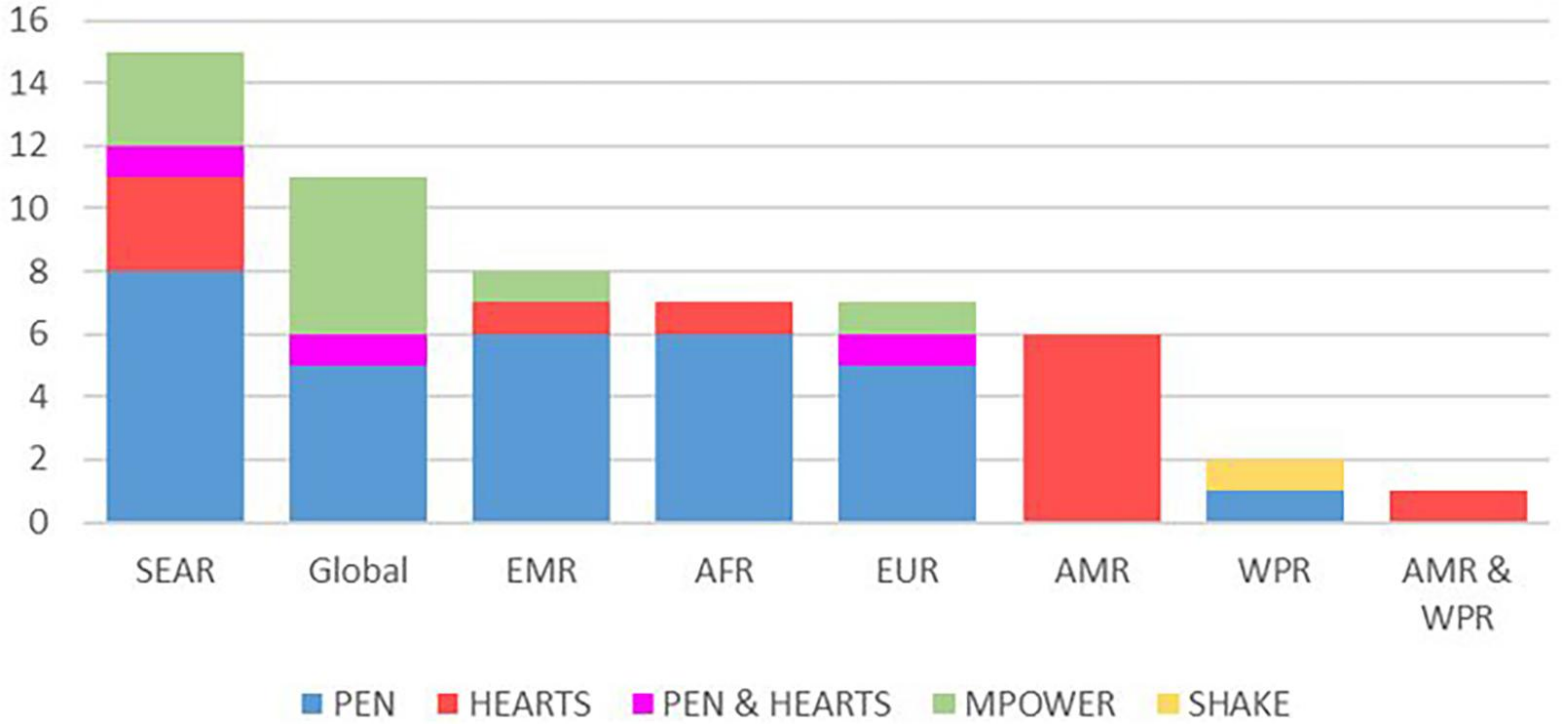
Studies by wHO region and technical package. AFR, African Region; AMR, Region of the Americas; EMR, Eastern Mediterranean Region; EUR, European Region; SEAR, South-East Asian Region; WPR, West Pacific Region.

Over half of the reviewed articles (*n* = 31) discussed the WHO PEN or WHO PEN-Plus technical packages. Twelve articles focused on the WHO HEARTS technical package (12, 13, 16–26), and 10 included the WHO MPOWER technical package (27–36). Of the remaining articles, three concerned an integrated WHO PEN and HEARTS technical package program (37, 38), and one concerned the WHO SHAKE technical package (37). Studies of HEARTS were concentrated in the Americas and South-East Asia Regions, while studies of MPOWER were largely conducted within the South-East Asia Region or at the Global level. PEN was by far the most studied technical package in the African, European, and Eastern Mediterranean Regions (Figure 2). Overall, the distribution of studies reflected regional implementation priorities. PEN and PEN-Plus predominated in settings focused on strengthening primary care for NCDs, whereas HEARTS was more commonly evaluated in countries implementing cardiovascular disease programmes, and MPOWER studies largely examined population-level tobacco control policies.

### Stage of implementation when research was conducted

**Figure 3:**
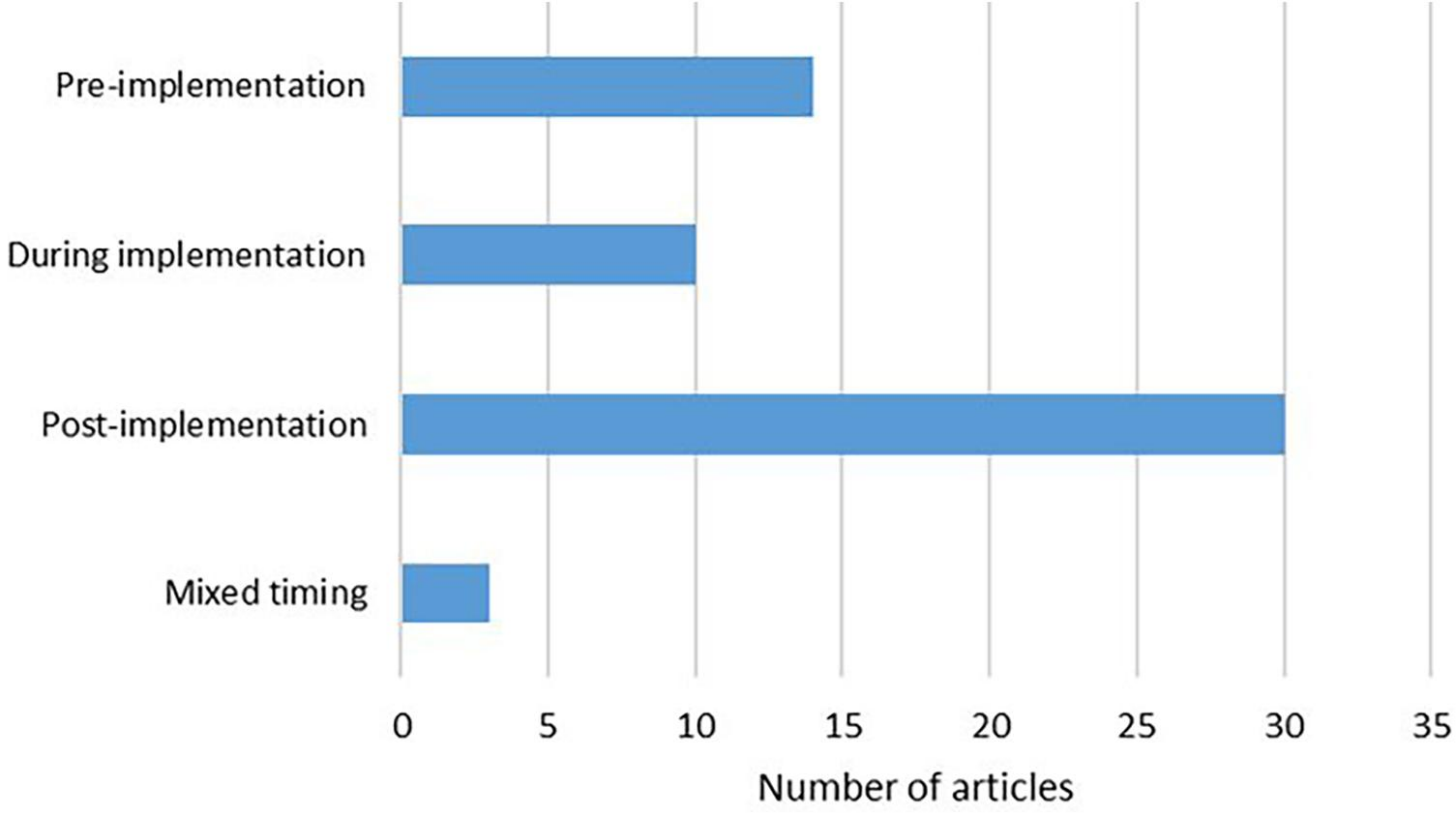
Stages of technical package implementation reported across studies.

The majority of studies (*n* = 30) were conducted post implementation of the package. They retrospectively evaluated the implementation of technical packages, their impact on population health, and the barriers and facilitators to successful implementation. For instance, AlHelo et al. (41) reviewed patient medical records and followed up with those same individuals for 2 years after PEN implementation in Gaza to determine whether implementation of the guidelines had impacted population blood pressure, BMI, and other NCD risk factors. Studies classified as “pre-implementation” (*n* = 14) generally assessed a country's health landscape or capacity to implement WHO NCD technical packages prior to national implementation. As an example, Aryal et al. (54) conducted a baseline assessment of training, diagnostics, equipment, and medicine supplies in Nepalese health facilities prior to implementation of PEN. Studies classified as “during implementation” (*n* = 10) were conducted concurrently with national efforts to implement technical packages, such as Bollars et al. (44), which identified population health status and lessons learned from a pilot implementation of WHO PEN disease and risk factor screening protocol in Samoa and analysed factors needed for scale up. Three articles were multi-country studies that evaluated the aspects and impacts of technical packages across multiple stages of implementation (Figure 3).

Across implementation stages, studies shifted in focus from assessing health system readiness before implementation to evaluating implementation processes and health outcomes during and after rollout. However, relatively few studies examined long-term maintenance, indicating that implementation research remains concentrated on early implementation rather than sustainability.

### Implementation science frameworks adopted

Only one of the studies included in this review explicitly used an implementation science framework; specifically, the Consolidated Framework for Implementation Research (CFIR) was applied by Giraldo (18) to map implementation strategies. Six studies applied other types of frameworks: the Health System Dynamics Framework by Kabir et al. (63); the Cabana framework by Albelbeisi et al. (53); the Consolidated Criteria for Reporting Qualitative Research by Aye et al. (55); the Heart Outcomes Prevention and Evaluation Program Framework by Khan et al. (21); the Primary Health Care Performance Initiative Conceptual Framework by Okoli et al. (24); and the WHO-reassured criteria by Bernabé-Ortiz et al. (56). These frameworks were used to assess the context of NCD service delivery, develop surveys and training tools, and provide guidelines for reporting findings.

Recognizing this diversity in theoretical approaches, we differentiated between studies that explicitly employed formal implementation science frameworks and those that used context-specific or pragmatic frameworks to guide their design or reporting. This distinction structured both data extraction and interpretation, acknowledging that some studies explicitly applied formal implementation frameworks (CFIR), while others used pragmatic or context-specific approaches.

Overall, the limited use of formal implementation science frameworks suggests that implementation research on WHO NCD technical packages has largely remained pragmatic and programme-driven, with relatively little application of established implementation theories to guide design or evaluation.

### Stakeholder engagement and interrelationships

Stakeholder engagement identifies stakeholders and involves them in program development, implementation, research and decision-making. Seventeen studies included some aspects of stakeholder engagement. Stakeholder groups engaged by these studies included health workers; program managers; civil society groups and media; and professional experts. Studies engaging these groups of stakeholders are listed in Table 2.

**Table 2:** Studies sorted by engagement with stakeholder groups.

Stakeholder group engaged	Studies by author
Health workers	Aryal et al. (54); Aye et al. (55); Bollars et al. (44); Collins et al. (59); Collins et al. (70); Gupta et al. (61); Kaur et al. (29); Krishna et al. (22); Laatikainen et al. (13); Okoli et al. (24); Philbert et al. (25); Tenzin et al. (38)
Program managers and implementors	Bollars et al. (44); Boudreaux et al. (57); Husain et al. (20); Giraldo (18); Okoli et al. (24)
Patients	Laatikainen et al. (14); Okoli et al. (24)
Civil society, media, and professional experts	Bollars et al. (44); Hurst et al. (11); Malhi et al. (33)

Three studies included stakeholders from multiple groups. Bollars et al. (44) included local stakeholders, health ministry, National Health Service representatives, local WHO Country Office Staff, and representatives from villages to ensure that the intervention was contextually appropriate and aligned with both community needs and national health priorities; Okoli et al. (24) included patients, non-physician health workers, administrators, and primary care physicians who provided critical inputs in tailoring the program to address both the clinical and operational realities of hypertension management in primary care setting; and Laatikainen et al. (13) included clinic managers, doctors, medical assistants, nurses, and patients to support engagement objectives. On top of incorporating diverse stakeholder perspectives, some studies conducted preliminary outreach to ensure that research findings would be relevant and useful to key stakeholder groups. Collins et al. (70), for instance, selected their study site based on a nomination from the Ministry of Health in Tajikistan.

Aligned with the ERIC strategy cluster “Develop stakeholder interrelationships,” these approaches reflect strategies such as building coalitions, involving consumers, and promoting network weaving to strengthen the implementation climate for NCD technical packages. Across studies, stakeholder engagement occurred at multiple levels: national (e.g., Ministry of Health, WHO Country Offices), facility (e.g., clinic managers, providers), and community (e.g., patients, local representatives) to enhance contextual relevance, promote ownership, and facilitate program adaptation.

Stakeholder engagement emerged as a consistent feature of successful implementation across settings, particularly where programmes required adaptation to local health system and community contexts. However, engagement was predominantly concentrated among health workers and programme managers, with comparatively limited involvement of patients and communities.

### Context assessment: barriers and facilitators to implementation

Situational analysis is crucial in implementation science as it provides the foundation for developing resource-appropriate strategies, ensuring that interventions are tailored to the entire continuum of care while considering the broader structural, sociocultural, and financial contexts. The included studies identified various barriers and facilitators to implementation, which are listed in Tables 3, 4, respectively.

**Table 3:** Categories of implementation barriers identified in the included studies.

Barriers identified	Studies by Author
Lack of essential medicines	AlHelo and Elessi (41); Giraldo et al. (19); Krishna et al. (22); Nyarko et al. (67); Okoli et al. (24); Tripathy and Mishra (51)
Lack of medical equipment and technologies	AlHelo and Elessi (41); Aryal et al. (54); Collins et al. (59); Tripathy and Mishra (51)
Poor incentives for healthcare providers	Albelbeisi et al. (53); Giraldo et al. (19)
Inadequate training of health workers	Aye et al. (55); Hurst et al. (11); Nsour et al. (23); Okoli et al. (24); Tripathy and Mishra (51)
Inadequate staffing and staff turnover	Nsour et al. (23); Collins et al. (59); Nyarko et al. (67); Ruderman et al. (69); Tenzin et al. (38); Tripathy and Mishra (51)
Lack of digitised and interoperable health systems	Bollars et al. (44); Giraldo et al. (19); Laatikainen et al. (48); Tripathy and Mishra (51)
Weak regulatory landscapes and industry interference	Giraldo et al. (19); Hurst et al. (11); Husain et al. (20)
Poor or absent screening programs	Hurst et al. (11); Laatikainen et al. (48)
Affordability, accessibility, and insurance coverage of NCD care	Ayat et al. (42); Boudreaux et al. (57); Hurst et al. (11); Laatikainen et al. (48)
Patient distrust in health system	Krishna et al. (22); Laatikainen et al. (48)

**Table 4:** Categories of implementation facilitators identified in the included studies.

Facilitators identified	Studies by Author
Community-based and participatory approaches	Bollars et al. (44)
Public awareness campaigns	Hurst et al. (11); Malhi et al. (33); Minh et al. (34)
Support from senior leadership in health care facilities and government	Nsour et al. (23); Philbert et al. (45)
Standardized treatment protocols	Brettler et al. (16); Collins et al. (59); Giraldo et al. (19)
Flexible interventions for local adaptation	Giraldo et al. (19); Hurst et al. (11)
Quality health data and performance monitoring	Brettler et al. (16); Giraldo et al. (19); Laatikainen et al. (48); Rattanavipapong et al. (68); Shariati et al. (50)
Subsidised costs for patients	Hyon et al. (46)
Continuity of care and follow-up in health systems	Aye et al. (55); Brettler et al. (16); Tripathy and Mishra (51); Wangchuk et al. (52)
Team-based care and task-shifting roles at primary health care facilities	Giraldo et al. (19)

From an implementation science perspective, these findings align closely with the ERIC strategy cluster “Use evaluative and iterative strategies,” which emphasizes identifying contextual determinants prior to and during implementation. The reported barriers (Table 3) such as lack of essential medicines, equipment, trained staff, and interoperable systems highlight gaps that can be addressed through ERIC strategies like assessing organizational readiness, providing training and technical assistance, and changing infrastructure to support delivery.

Similarly, identified facilitators reflect the application of ERIC strategies under clusters such as “Adapt and tailor to context” and “Train and educate stakeholders.” Community-based and participatory approaches, public awareness campaigns, and support from senior leadership build stakeholder buy-in and adapt interventions to local conditions. Structural facilitators such as standardized protocols, quality data systems, and team-based models of care align with strategies to change infrastructure and revise professional roles, promoting sustainability and integration within health systems.

Across technical packages, the most frequently reported barriers were health system constraints, including workforce shortages, limited access to medicines and diagnostics, weak health information systems, and financing challenges, suggesting that implementation barriers were largely systemic rather than package-specific. Conversely, successful implementation was consistently associated with leadership support, standardized protocols, capacity building, and context-specific adaptation.

### Health system capacity and infrastructure for implementation

Assessing health system capacity for implementing interventions is critical in implementation science because it directly influences the feasibility, scalability, and sustainability of interventions. Eighteen studies reported on the existing capacity within the relevant health system to implement WHO packages. These included assessments of the capacity for guideline adherence and implementation [Albelbeisi et al. (53); Bernabe-Ortiz et al. (56)], service availability [Boudreaux et al. (57); Okoli et al. (24); Bollars et al. (78)], health facility assessment [Aye et al. (55); Mutale et al. (66)], policy analysis [Calikoglu et al. (27)], identifying barriers and challenges to package implementation (Flood et al. (17); Giraldo et al. (19); Hurst et al. (11); Laatikainen et al. (47); Nsour et al. (23)), testing package through pilot implementation [Hyon et al. (46)] and suitability assessment [Campbell et al. (12)].

These activities collectively reflect implementation strategies from the ERIC clusters “Use evaluative and iterative strategies” and “Change infrastructure,” emphasizing efforts to assess readiness, identify gaps, and adapt health system structures before or during rollout of WHO technical packages.

In addition, we reviewed the studies for capacity building efforts before package implementation. Of all studies, 47 included at least one of the five types of capacity-building strategies: training, tools, technical assistance, incentives, and assessment or feedback components. Seventeen studies included training of health care workers (*n* = 14), of trainers (*n* = 5), or a combination of health care workers and trainers (*n* = 2). [(11–13, 16, 18–20, 23, 25, 41, 44, 46, 52, 53, 55, 58, 69)] Sixteen of the studies reported on various tools, comprising clinical decision support tools (*n* = 7), clinical and diagnostic tools (*n* = 5), the HEARTS costing tool (*n* = 2), scorecards (*n* = 1), and WHO/ISH (International Society of Hypertension) charts (*n* = 1) (11–13, 16–18, 20, 44, 46, 48, 50, 58–62). Of the 11 studies that reported on the use of technical assistance, 4 were connected to partnerships between multilateral and local organization, 3 between local partners, 2 between unknown partners, 1 between the government and a local partner, and 1 between a non-governmental organization and a local partner (12, 17–19, 38, 44, 46, 48, 52, 58, 69). None of the studies reported on the use of incentives for capacity building.

These findings map onto ERIC strategies such as “Train and educate stakeholders,” “Provide ongoing consultation,” and “Develop and distribute educational materials.” The reliance on technical assistance and use of standardized tools highlight system-level mechanisms to institutionalize learning and strengthen implementation infrastructure. The lack of incentives as a strategy suggests an underutilized area for improving provider engagement and retention in NCD programs.

Taken together, these findings indicate that implementation efforts were primarily directed towards strengthening service delivery capacity through training, technical assistance, and standardized tools, while comparatively less attention was given to financial or organizational incentives that may support long-term implementation.

### Implementation outcomes and evaluative strategies

**Figure 4:**
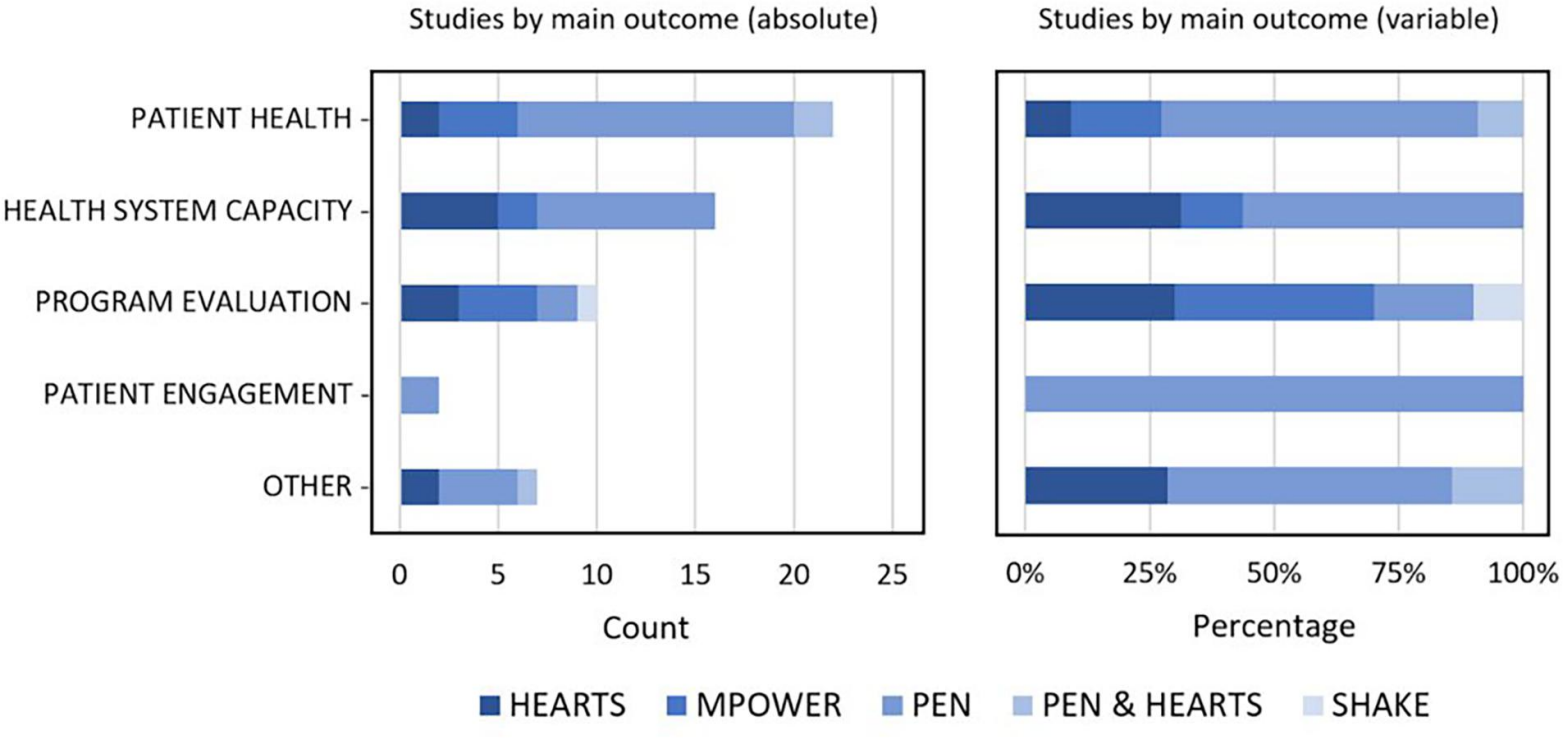
Main outcomes by WHO technical package.

Patient related outcomes: Across the included studies, outcomes were reported at multiple levels: patient health, patient engagement, and system-level measures which correspond to ERIC's cluster “Use evaluative and iterative strategies,” reflecting the use of feedback, readiness assessments, and performance monitoring during implementation. Twenty-four studies reported on patient-related outcomes (22 on patient health, 2 on patient engagement). Of the 24, 16 included the PEN package (41–52), 4 the MPOWER package (30, 32, 35, 36), 2 the HEARTS package (20, 25), and 2 both the PEN and HEARTS packages (38). Figure 4 displays the number of each main outcome grouped by WHO technical package.

Studies linked patient health outcomes to implementation of the PEN package, including improvements in cardiovascular health indicators, decreases in the incidence rate of myocardial infarction and stroke, reductions in alcohol and tobacco use, increases in levels of screening for NCDs and NCD risk factors, and reductions in mortality. As an example, Laatikainen et al. (47) found that Moldovan patients receiving treatment in clinics that implemented WHO PEN guidelines achieved improved HbA1c and hypertension control compared to patients at other clinics. Studies that analysed the MPOWER package showed patient health outcomes relating to decreases in tobacco use and reductions in smoking-attributable deaths (30, 32, 35, 36). For instance, Ngo et al. (35) observed that more comprehensive MPOWER implementation across more than 60 countries led to lower prevalence of smoking and lower average cigarette consumption, and Levy et al. (32) found that stronger health warnings recommended by MPOWER had prevented over 13 million smoking-attributable deaths across 43 countries between 2014 and 2016. The two studies that measured patient health outcomes following implementation of the HEARTS package found that patients increased control of their blood pressure after program implementation (23, 25). Lastly, one study focused on implementation of both the PEN and HEARTS packages discovered that they led to higher overall NCD control (38).

Of the two studies that focused on outcomes related to patient engagement, both reported increased levels of screening for NCDs and NCD risk factors. Bollars et al. (44) evaluated screening levels after four protocols of the WHO PEN package were implemented in Samoa (44). This study reported that 6.7% of participants older than 40 years were identified as being at a high-risk level for at least one NCD and were referred to further treatment. Tripathy and Mishra (51) found that more than 90% of the target population were screened for NCDs and NCD risk factors after PEN implementation in Samoa (51).

Assessment and feedback outcomes: Nine studies used assessment and feedback tools as a means of evaluation. The most common assessment and feedback strategies were patient feedback (*n* = 2), assessments of a health facilities' readiness to implement an intervention (*n* = 2), feedback on barriers to patient adherence (*n* = 1), feedback on curriculum uptake (*n* = 1), patient and provider feedback (*n* = 1), performance assessments (*n* = 1), and quality care feedback (*n* = 1) (21, 22, 38, 46, 48, 53–55, 61). For instance, Aryal et al. (54) applied a cross-sectional study to assess health facilities' readiness for PEN implementation. Meanwhile, Khan et al. (21) used an iterative and phased approach to develop, test, and implement a curriculum on CVD prevention and control in Colombia, Malaysia, and low-resource regions of Canada.

This pattern reflects ERIC strategies within the cluster “Use evaluative and iterative strategies”, showing the use of feedback loops and readiness assessments to refine interventions during implementation. The emphasis on patient-level and facility-level outcomes also indicates multi-level measurement — a critical aspect of understanding how implementation fidelity and effectiveness interact.

Across studies, implementation success was most commonly assessed using patient health outcomes and facility readiness measures, whereas implementation outcomes such as adoption, fidelity, acceptability, and sustainability were reported much less frequently. This highlights an important gap between programme evaluation and implementation science evaluation.

Other System-Level Outcomes: Seven studies produced findings which were not possible to categorise as patient health, patient engagement, health system capacity, or program evaluation outcomes. These “other” outcomes were largely measured at the system level and skewed towards qualitative methods that review barriers and facilitators, challenges and opportunities, and technical package implementation status. For example, Muratalieva et al. (65) reviewed several national NCD responses and their implementation of PEN, and Hanley (62) assessed the status of integration of HIV within CVD prevention and management guidelines in South Africa. Hurst et al. (11) identified global challenges to the implementation of COPD guidelines, while Flood et al. (17) described opportunities to integrate diabetes into the HEARTS package.

Table 5 lists all 57 studies assessed in this review, identifying them first by their authors and year of publication. The table then shows the specific geographic area and technical package that are studied, and whether the effects of any capacity building approaches were assessed. The final two columns categorise the measured outcomes and the levels at which those outcomes were measured. Outcomes were most commonly measured at the national level, although many studies—particularly those measuring health system capacity—examined outcomes at the system level, a context-specific scope that adheres more often to networks of facilities than other established geographic boundaries or administrative units. These studies demonstrate that implementation research extended beyond clinical effectiveness to examine policy integration, governance, and health system organization, reflecting the complex, multisectoral nature of implementing WHO technical packages.

**Table 5:** Descriptive characteristics of included studies.

Authors & Publication Year	Country/Region	WHO Technical Package	Capacity Building (yes/no)	What was measured	Level of measurement
Albelbeisi et al. (53)	Palestine	PEN	Yes	Health system capacity	System level
AlHelo and Elessi (41)	Palestine	PEN	Yes	Patient health	Patient level
Aryal et al. (54)	Nepal	PEN	No	Health system capacity	Facility level
Ayat et al. (42)	Iran	PEN	No	Patient health	National level
Aye et al. (55)	Myanmar	PEN	Yes	Program evaluation	Patient level
Basu et al. (43)	South Africa	PEN	No	Patient health	National level
Bernabé-Ortiz et al. (56)	Global	PEN	No	Other (Challenges surrounding access to diagnostics and monitoring tools from the WHO)	System level
Bollars et al. (44)	Samoa	PEN	Yes	Patient engagement	Patient level
Boudreaux et al. (57)	Africa	PEN-Plus	No	Health system capacity	System level
Brettler et al. (16)	Latin America	HEARTS	Yes	Other (Identification of key drivers of HTN control)	System level
Calikoglu et al. (27)	Türkiye	MPOWER	Yes	Program evaluation	National level
Campbell et al. (12)	Latin America	HEARTS	Yes	Program evaluation	National level
Collins et al. (70)	Tajikistan	PEN & HEARTS	Yes	Patient Health	Facility Level
Collins et al. (58)	Moldova	PEN	Yes	Patient Health	Facility level
Collins et al. (59)	Jordan	PEN	No	Program evaluation	Patient level
Dukpa et al. (45)	Bhutan	PEN	No	Patient health	National level
Flood et al. (17)	Latin America	HEARTS	Yes	Other (Description of opportunities for integrating DM clinical care and policy within HEARTS HTN framework)	System level
Flood et al. (60)	Global	PEN	No	Patient Health	National level
Giraldo (18)	Colombia	HEARTS	Yes	Health system capacity	National level
Giraldo et al. (19)	Latin America	HEARTS	Yes	Health system capacity	Regional level
Gupta et al. (61)	India	PEN	No	Health system capacity	System level
Hanley (62)	South Africa	PEN	No	Other (Status of integration of HIV management and CVD prevention and management guidelines in South Africa)	National level
Heydari (28)	Middle East	MPOWER	No	Program evaluation	Regional level
Hurst et al. (11)	Global	PEN	Yes	Other (Challenges to implementation of CPOD guidelines)	System level
Husain et al. (20)	Thailand	HEARTS	Yes	Program evaluation	District level
Husain et al. (31)	Global	MPOWER	No	Health system capacity	National level
Hyon et al. (46)	North Korea	PEN	Yes	Patient health	Patient level
Jarvis et al. (37)	Global	PEN & HEARTS	No	Other (Review of national EMLs for their inclusion of priority NCD interventions recommended by the WHO)	National level
Kabir et al. (63)	Bangladesh	PEN	No	Health System Capacity	System level
Kaur et al. (29)	South-East Asia	MPOWER	No	Health system capacity	National level
Khan et al. (21)	Canada, Colombia, and Malaysia	HEARTS	No	Health system capacity	Provider level
Krishna et al. (22)	India	HEARTS	No	Health system capacity	Provider level
Laatikainen et al. (47)	Moldova	PEN	No	Patient health	Facility level
Laatikainen et al. (13)	Ukraine	PEN	Yes	Patient health	System level
Laatikainen et al. (48)	Kyrgyz Republic	PEN	No	Patient health	Patient level
Levy et al. (30)	Global	MPOWER	No	Patient health	National level
Levy et al. (32)	Global	MPOWER	No	Patient health	National level
Malhi et al. (33)	India	MPOWER	No	Program evaluation	National level
Minh et al. (34)	Vietnam	MPOWER	No	Program evaluation	National level
Mirzaei and Mirzaei (64)	Iran	PEN	No	Patient health	Patient level
Muratalieva et al. (65)	Eurasia	PEN	No	Other (Review at the national level to their response to NCDS and the implementation of the WHO PEN disease interventions for primary health care)	National level
Mutale et al. (66)	Zambia	PEN	No	Health system capacity	Facility level
Ngo et al. (35)	Global	MPOWER	No	Patient health	National level
Nsour et al. (23)	Jordan	HEARTS	Yes	Patient health	Patient level
Nyarko et al. (67)	Ghana	PEN	No	Health system capacity	Facility level
Okoli et al. (24)	Nigeria	HEARTS	No	Health system capacity	System level
Peiris et al. (49)	Global	PEN	No	Patient health	National level
Philbert et al. (25)	Saint Lucia	HEARTS	Yes	Patient health	Facility level
Pidugu et al. (26)	Bangladesh	HEARTS	No	Program evaluation	National level
Rattanavipapong et al. (68)	Indonesia	PEN	No	Health system capacity	National level
Ruderman et al. (69)	Malawi	PEN	Yes	Health system capacity	Provider level
Shahrir et al. (39)	Malaysia	SHAKE	No	Program evaluation	National level
Shariati et al. (50)	Iran	PEN	No	Patient health	Patient level
Song et al. (36)	Global	MPOWER	No	Patient health	National level
Tenzin et al. (38)	Bhutan	PEN & HEARTS	No	Patient health	System level
Tripathy and Mishra (51)	Global	PEN	No	Patient engagement	System level
Wangchuk et al. (52)	Bhutan	PEN	Yes	Patient health	Facility level

### Sustainability and long-term maintenance

Among all studies that focused on the post-implementation stage (*n* = 30), only two studies (Collins et al. (58); Heydari et al. (28) had a sustainability component. Sustainability is defined as “the extent to which an evidence-based intervention can deliver its intended benefits over an extended period of time after external support is terminated.” (71) In line with this definition, Collins et al. showcased significant reduction in the prevalence of normal blood pressure and an increase in the proportion of patients with hypertension who achieved blood pressure targets after two years of package implementation. Heydari et al. demonstrated improvements in monitoring prevalence data within EMR countries after 10 years of MPOWER implementation (28, 70).

The scarcity of studies evaluating sustainability indicates that long-term maintenance remains one of the least studied aspects of WHO technical package implementation in LMICs. Most evaluations focused on implementation or short-term outcomes, limiting understanding of whether implementation gains are sustained once external support or initial programme funding ends.

## Discussion

This scoping review aimed to evaluate the application of implementation science to the adoption, implementation, and evaluation of WHO technical packages for NCDs in LMICs, and to summarise barriers and facilitators. The findings reveal that implementation science frameworks are recognised as promising tools for improving health outcomes. However, the NCD policy field lacks consistent application of implementation science methods and clear outcome definitions. This inconsistency may compromise intervention sustainability and broader utility. Our results also show that implementation science has been applied unevenly across different types of WHO technical packages. Service-delivery packages (such as PEN, PEN-Plus, and HEARTS) often rely on clinical implementation processes. In contrast, regulatory packages (such as MPOWER, SHAKE, SAFER, and REPLACE) require approaches that consider policy adoption, governance, and enforcement. However, few studies explicitly engaged with frameworks suited to these implementation pathways. This is notable because more than two hundred implementation science frameworks are now available globally. These frameworks provide structured tools to guide planning, adaptation, evaluation, and learning.

Among the studies included in this review, implementation science frameworks were infrequently utilized, and implementation outcomes were not always clearly defined or measured. The field is still emerging (40), and some delay in integrating implementation science concepts into practice and published research is expected. Nevertheless, there remains a critical gap in sustainability assessments (9). Only two studies included explicit sustainability analyses, despite sustainability being central to the long-term impact of both clinical and regulatory interventions. This underscores the need for robust methodologies that ensure interventions can be maintained long-term. This review reinforces the need to build structured approaches to NCD implementation science, and advocates for systematic capacity-building and planning to advance the field and scale up effective interventions. Included studies acknowledged that NCD policy research requires funding that reflects the complexity and time needed for contextualization. However, funding should also align with policy and implementation cycles. Additionally, the field requires investment in multidisciplinary research capacity and leadership. It also needs stronger networks to encourage cohesive action and align NCD-related scale-up research activities globally (72).

The limited and inconsistent application of implementation science frameworks observed in this review may partly explain the variability in implementation planning, evaluation, and reporting across studies. Established frameworks such as EPIS (Exploration, Preparation, Implementation, Sustainment) emphasise implementation as a dynamic process influenced by both inner and outer contextual factors, making them particularly relevant for adapting WHO technical packages across diverse LMIC settings. Likewise, RE-AIM (Reach, Effectiveness, Adoption, Implementation, and Maintenance) encourages evaluation beyond intervention effectiveness by considering implementation, adoption, and long-term maintenance, domains that were infrequently assessed in the included studies. The inconsistent definition and measurement of implementation outcomes identified in this review also highlight the value of Proctor's Implementation Outcomes Framework, which provides standardised constructs such as acceptability, adoption, appropriateness, feasibility, fidelity, implementation cost, penetration, and sustainability. Greater use of these complementary frameworks could improve methodological consistency, facilitate comparison across studies, and strengthen the evidence base for implementing and scaling WHO technical packages for NCDs in LMICs.

The remainder of this discussion draws on the broader public health and policy literature to consider funding, governance, and institutional factors relevant to implementation science. While informed by the findings above, these points reflect broader contextual considerations and recommendations rather than conclusions derived directly from the studies included in this review.

Political commitment and sustainable funding mechanisms remain critical for scaling up implementation science and addressing NCDs effectively. Up to the time of writing, several funders, such as the National Institute for Health (United States), National Institute for Health Research (United Kingdom), and the Global Alliance for Chronic Diseases, have been funding research on implementation science and NCDs, supporting and advancing the field. However, shifts in global funding landscapes may affect the continuity and expansion of such investments. This includes changes in ODA and broader research financing seen in early 2025. These developments underscore the need for renewed and sustained commitment.

Despite the potential of implementation science to transform service delivery, funding for such research often relies on external donors. Domestically driven investments in research capacity are essential, alongside career incentives and platforms that connect findings with policy dialogues (73). Therefore, more attention needs to be paid to power imbalances (e.g., funding, international interests, commercial influences, etc.) in research ecosystems. Such dynamics are particularly relevant for regulatory packages, where commercial determinants and political economy factors heavily shape implementation feasibility.

The field must advance beyond its current fragmented state to build greater capacity for conducting implementation science within LMICs, involving multidisciplinary teams to enhance integration and scalability (9). Several challenges remain, such as misaligned incentives, limited resources, and the complexity of engaging stakeholders. To address these challenges, collaborative approaches involving policymakers, healthcare providers, and communities are needed. These collaborations can address power dynamics and tailor interventions to local contexts (74).

Several tools and guides are available to support researchers and programme implementers in conducting implementation research in public health, and specifically for NCDs (75, 76). The literature signals a need for methodological innovation and alignment of research and policy cycles. Successful examples from countries illustrate the potential of implementation science when embedded in policy processes. Embedded implementation research integrates research methods and approaches within existing health programme implementation and policymaking cycles. This approach can maximise the benefits of implementation research and delivery science (77). Embedding implementation science within ministries of health and regulatory institutions may be particularly valuable for accelerating uptake and ensuring that global guidance is adapted in ways that are institutionally feasible and politically acceptable. Future efforts should integrate implementation science more fully into health system development, with greater emphasis on co-creation of knowledge and iterative feedback loops.

### Limitations

This review has several limitations that must be acknowledged. First, we included only peer-reviewed articles written in English, potentially excluding relevant studies in other languages or unpublished works, which could have provided additional insights. Second, the reliance on self-reported use of implementation science frameworks may have led to an overestimation of their actual application and underreporting of other relevant methodologies. Third, the geographic representation of the studies was uneven, with certain regions and countries, particularly low-income settings, being underrepresented. This limits the generalizability of the findings across diverse LMIC contexts.

In addition, our review focused on published evidence related to a defined set of WHO technical packages. While appropriate and necessary for maintaining clarity and methodological rigour, this approach may not have captured other relevant implementation programmes, including initiatives led by NGOs or other providers, as well as newer programmes and emerging literature published in recent years. As a result, there may be valuable implementation experiences occurring in LMIC settings that were not reflected in our analysis. However, this focus also strengthens the policy relevance of the review: these WHO technical packages are formally endorsed, widely disseminated, and actively shaping government responses to NCDs in many LMICs, making them particularly important to understand from an implementation perspective.

The absence of a formal quality assessment of the included studies restricts the ability to ascertain the robustness of the evidence. The findings should therefore be interpreted as a descriptive overview of the existing literature and its gaps. Finally, the review identified only a few studies focusing on sustainability assessments and long-term outcomes, which are critical for evaluating the long-term impact of WHO technical package implementation. These gaps underline the need for future implementation science studies to adopt more standardized frameworks and incorporate sustainability and policy relevance into their design.

## Conclusions

Despite progress in applying implementation science to NCDs, the field faces challenges in navigating an overwhelming number of frameworks and theories leading to inconsistent application. Additionally, limited focus on sustainability, and insufficient stakeholder collaboration continue to hinder effective policy implementation. Addressing these challenges requires structured approaches, capacity building, and stronger integration of research into policy making. Increased political commitment, sustainable funding, and mechanisms for knowledge translation are essential to bridge the gap between evidence and practice. Advancing implementation science can significantly enhance the scalability and impact of NCD interventions, contributing to global health equity and universal health coverage. Following the adoption in December 2025 of the Political Declaration at the Fourth High-level Meeting of the UN General Assembly on the Prevention and Control of NCDs, there is renewed global commitment and a critical window to embed implementation research within national strategies, ensuring that commitments are translated into concrete policy action and measurable impact.
