# Supplementary material for: Using implementation science for WHO noncommunicable disease technical packages in low-and middle-income countries: a scoping review
Source: Front Health Serv. 2026 Jul 30;6:1816601. doi: 10.3389/frhs.2026.1816601 (PMC13470048; doi:10.3389/frhs.2026.1816601)
Supplement: Supplementary file 1 [file Table1.docx]

**Supplement 1**

**PubMed Search Strategy**

("Afghanistan"[tiab] OR "Albania"[tiab] OR "Algeria"[tiab] OR "Angola"[tiab] OR "Argentina"[tiab] OR "Armenia"[tiab] OR "Azerbaijan"[tiab] OR "Bangladesh"[tiab] OR "Belarus"[tiab] OR "Belize"[tiab] OR "Benin"[tiab] OR "Bhutan"[tiab] OR "Bolivia"[tiab] OR "Bosnia and Herzegovina"[tiab] OR "Botswana"[tiab] OR "Brazil"[tiab] OR "Bulgaria"[tiab] OR "Burkina Faso"[tiab] OR "Burundi"[tiab] OR "Cambodia"[tiab] OR "Cameroon"[tiab] OR "Cape Verde"[tiab] OR "Central African Republic"[tiab] OR "Chad"[tiab] OR "China"[tiab] OR "Colombia"[tiab] OR "Comoros"[tiab] OR "Congo"[tiab] OR "Costa Rica"[tiab] OR "Côte d'Ivoire"[tiab] OR "Cuba"[tiab] OR "Democratic Republic of the Congo"[tiab] OR "Djibouti"[tiab] OR

"Dominica"[tiab] OR "Dominican Republic"[tiab] OR "Ecuador"[tiab] OR "Egypt"[tiab] OR "El Salvador"[tiab] OR "Equatorial Guinea"[tiab] OR "Eritrea"[tiab] OR "Eswatini"[tiab] OR "Ethiopia"[tiab] OR

"Fiji"[tiab] OR "Gabon"[tiab] OR "Gambia"[tiab] OR "Georgia"[tiab] OR "Ghana"[tiab] OR "Grenada"[tiab] OR "Guatemala"[tiab] OR "Guinea"[tiab] OR "Guinea-Bissau"[tiab] OR "Guyana"[tiab] OR "Haiti"[tiab] OR "Honduras"[tiab] OR "India"[tiab] OR "Indonesia"[tiab] OR "Iran"[tiab] OR "Iraq"[tiab] OR "Jamaica"[tiab] OR "Jordan"[tiab] OR "Kazakhstan"[tiab] OR "Kenya"[tiab] OR "Kiribati"[tiab] OR "Kosovo"[tiab] OR

"Kyrgyzstan"[tiab] OR "Lao PDR"[tiab] OR "Lebanon"[tiab] OR "Lesotho"[tiab] OR "Liberia"[tiab] OR "Libya"[tiab] OR "Madagascar"[tiab] OR "Malawi"[tiab] OR "Malaysia"[tiab] OR "Maldives"[tiab] OR "Mali"[tiab] OR "Mauritania"[tiab] OR "Mauritius"[tiab] OR "Mexico"[tiab] OR "Moldova"[tiab] OR

"Mongolia"[tiab] OR "Montenegro"[tiab] OR "Morocco"[tiab] OR "Mozambique"[tiab] OR "Myanmar"[tiab] OR "Namibia"[tiab] OR "Nepal"[tiab] OR "Nicaragua"[tiab] OR "Niger"[tiab] OR "Nigeria"[tiab] OR "North Macedonia"[tiab] OR "Pakistan"[tiab] OR "Papua New Guinea"[tiab] OR "Paraguay"[tiab] OR "Peru"[tiab] OR "Philippines"[tiab] OR "Rwanda"[tiab] OR "Samoa"[tiab] OR "São Tomé and Príncipe"[tiab] OR "Senegal"[tiab] OR "Serbia"[tiab] OR "Sierra Leone"[tiab] OR "Solomon Islands"[tiab] OR "Somalia"[tiab] OR "South Africa"[tiab] OR "South Sudan"[tiab] OR "Sri Lanka"[tiab] OR "Sudan"[tiab] OR "Suriname"[tiab] OR "Syria"[tiab] OR "Tajikistan"[tiab] OR "Tanzania"[tiab] OR "Thailand"[tiab] OR "Timor-Leste"[tiab] OR "Togo"[tiab] OR "Tonga"[tiab] OR "Tunisia"[tiab] OR "Turkey"[tiab] OR "Turkmenistan"[tiab] OR "Uganda"[tiab] OR "Ukraine"[tiab] OR "Uzbekistan"[tiab] OR "Vanuatu"[tiab] OR "Venezuela"[tiab] OR

"Vietnam"[tiab] OR "Yemen"[tiab] OR "Zambia"[tiab] OR "Zimbabwe"[tiab] OR "low-income countr*"[tiab] OR "middle-income countr*"[tiab] OR "LMIC*"[tiab] OR "developing countr*"[tiab])

AND

("PEN"[tiab] OR "PEN Plus"[tiab] OR "PEN-Plus"[tiab] OR "HEARTS"[tiab] OR "MPOWER"[tiab] OR "SHAKE"[tiab] OR "SAFER"[tiab] OR "REPLACE"[tiab] OR "Package of Essential Noncommunicable Disease Interventions"[tiab])

AND

("implementation science"[tiab] OR implementation[tiab] OR dissemination[tiab] OR "policy analysis"[tiab] OR monitoring[tiab] OR evaluation[tiab] OR scale-up[tiab] OR adoption[tiab] OR

fidelity[tiab] OR sustainability[tiab])

AND

("2009/01/01"[Date - Publication] : "2022/12/31"[Date - Publication])
